# CT-based lung ventilation metrics: reference ranges and pulmonary function test correlations in healthy individuals

**DOI:** 10.1007/s00330-026-12559-8

**Published:** 2026-04-28

**Authors:** Charlotte E. van den Berg, Julian Dittrich, Sarah C. Scharm, Cornelia Schaefer-Prokop, Sabine Dettmer, Anna Hunkemoeller, Jan Eckstein, Julian Glandorf, Frank K. Wacker, Gesa H. Pöhler, Hoen-oh Shin

**Affiliations:** 1 Institute of Diagnostic and Interventional Radiology, Hannover Medical School, Hannover, Germany; 2 Biomedical Research in Endstage and Obstructive Lung Disease Hannover (BREATH), German Center for Lung Research, Hannover, Germany; 3 Department of Radiology, Radboud University, Nijmegen, The Netherlands; 4 Department of Radiology, Meander Medical Center, Amersfoort, The Netherlands; 5 Institute for Radiology, Nuclear Medicine, and Molecular Imaging, Heart and Diabetes Centre North Rhine-Westphalia, Ruhr University Bochum and Medical Faculty OWL (University of Bielefeld), Bad Oeynhausen, Germany; 6 Department of Radiology, University of Muenster, Muenster, Germany

**Keywords:** Thorax, Lung, Tomography (x-ray computed), Lung volume measurements, Pulmonary ventilation

## Abstract

**Objectives:**

CT-derived assessment of regional ventilation is possible with paired inspiration and expiration scans. Normative reference values for CT-derived ventilation remain undefined. The aim of this study is to establish reference values for pulmonary ventilation using CT, with pulmonary function tests (PFTs) as the clinical standard.

**Materials and methods:**

In this prospective, single-center study (December 2022–April 2024), 103 healthy adults underwent spirometry-guided inspiratory and expiratory CT. Lobes were segmented automatically using TotalSegmentator. Voxel-wise ventilation was quantified as the relative air volume change between inspiration and expiration via nonlinear registration, normalized to inspiratory lung volume. CT-derived lung volumes were compared with PFT-derived total lung capacity (TLC), residual volume (RV), and vital capacity (VC). Reference intervals were reported as mean ± standard deviation and 5th–95th percentiles. Multivariable analyses assessed the effects of sex, age, height, lung region, and gravitational orientation.

**Results:**

Ninety-one participants (mean age, 53 ± 12 years; 49 men) were included. Mean ventilation was 59.5% ± 8.5% (5th–95th percentile, 42.1–72.5%). CT-derived volumes were systematically lower than PFT values but strongly correlated: CT-TLC (−13.8%, *r* = 0.89), CT-RV (−2.5%, *r* = 0.80), and CT-VC (−20.8%, *r* = 0.82). Ventilation decreased with age (*p* < 0.001) and increased with height (*p* < 0.05). Regionally, ventilation was higher in the lower lobes (*p* < 0.003) and posterior regions (*p* < 0.001).

**Conclusion:**

CT-derived ventilation correlates strongly with PFT-indices but yields lower absolute values. Demographic and anatomical factors significantly influence ventilation distribution. These normative data provide a reference framework for clinical functional lung assessment.

**Key Points:**

***Question*** *Can normative reference values for CT-derived regional lung ventilation be established, and how do these metrics relate to pulmonary function tests and anatomical/demographic factors?*

***Findings*** *Mean CT-derived ventilation was 59.5% ± 8.5% with strong pulmonary function test correlations. Ventilation exhibited significant regional heterogeneity and systemic variations by anatomical location, age, and sex.*

***Clinical relevance*** *Normative reference values enable objective classification of CT-derived ventilation, standardizing interpretation and reducing subjectivity. They facilitate earlier detection of regional dysfunction, quantitative phenotyping, risk stratification, longitudinal monitoring, and comparability with pulmonary function testing and other imaging.*

**Graphical Abstract:**

**Figure:**
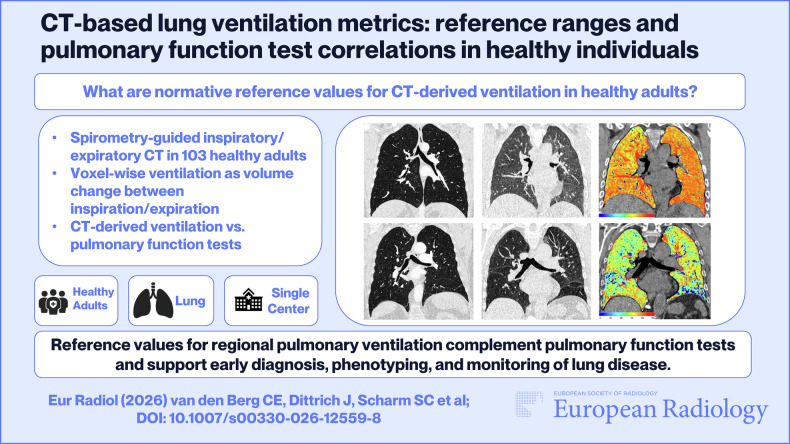

## Introduction

High-resolution computed tomography is the imaging modality of choice for assessing structural changes in lung diseases such as pulmonary fibrosis and chronic obstructive pulmonary disease [1]. Recent advances in CT technology and image post-processing have expanded its role beyond morphology, enabling quantitative assessment of functional impairment [1, 2].

Pulmonary function depends on effective ventilation—defined as the change in air volume between inspiration and expiration—ensuring gas exchange between alveoli and pulmonary capillaries. Traditional pulmonary function tests (PFTs) measure global indices of lung function but do not capture regional heterogeneity. Imaging-based biomarkers of ventilation and perfusion have therefore been developed using PET [3], SPECT [4], MR [5] with and without hyperpolarized gas, and 4D CT [6]. These methods provide spatially resolved functional information and have shown utility in both research and early clinical applications.

As CT-based functional lung imaging emerges as a clinically viable tool, the absence of normative reference values for ventilation limits its translation. Establishing such values in healthy adults is a prerequisite for distinguishing physiologic variation from disease-related abnormalities and for supporting individualized diagnosis and monitoring [7].

This study aimed to define normative reference values for ventilation parameters in a healthy adult population. Photon-counting CT was used as the imaging platform for this cohort. However, the quantitative ventilation assessment is transferable to any CT system and is not dependent on detector-specific features.

## Materials and methods

### Study design and participants

This prospective, single-center cross-sectional study (December 2022–April 2024) was approved by the local ethics committee (No. 10077_BO_S_2021StrSchVO) and the national radiation protection authority. Written informed consent was obtained from all participants.

A total of 103 healthy volunteers were recruited. Inclusion criteria were age 30–80 years and normal pulmonary status confirmed by medical history and physical examination. To minimize bias, 85.4% of participants underwent photon-counting CT and PFT on the same day; the remaining individuals were examined within 198–479 days (median, 327 days). Four participants (4.4%) were active smokers. Subjects with ≤ 5 pack-years and no structural or functional abnormalities were included. Exclusion criteria included pregnancy, recent respiratory infection, and contraindications to contrast media.

### Pulmonary function testing

Body plethysmography examinations were conducted in a dedicated pulmonary function laboratory by certified technicians adhering to the American Thoracic Society and European Respiratory Society guidelines [8, 9]. Measurements included quantification of total lung capacity (TLC), vital capacity (VC), and residual volume (RV).

### CT data acquisition

Examinations were performed on a Naeotom Alpha scanner (Siemens Healthineers) during spirometry-guided maximal inspiration and expiration (CTDIvol = 4.0 mGy per scan). Participants received detailed breathing instructions and underwent pre-examination training to ensure optimal compliance.

To enable comprehensive functional assessment, including perfusion and ventilation-to-perfusion ratio analyses, and to avoid redundant scanning, participants received 50 mL of iodinated contrast (Iomeron 400, Bracco), although only virtual non-contrast reconstructions were used for ventilation analysis. Perfusion analyses from this dataset will be reported separately. For ventilation assessment, virtual non-contrast images were reconstructed for both respiratory phases using dedicated software (syngo.via® v5.1, Siemens Healthineers). Images were reconstructed with ADMIRE iterative reconstruction, soft tissue kernel (Qr40), and 1 mm section thickness and 0.7 mm section interval (Table 1) [10].

**Table 1:** Photon-counting CT protocol

Parameter	Photon-counting CT protocol
Patient position	Supine
Series information	Two series, one in inspiration and one in expiration (5-min delay)
Scan location	Chest
Scanning plane	Transverse
Scan and display FOV range (cm) (adapted to participant size)	28–40
Image quality level	67
QIR level	2
Tube voltage (kV)	120
Gantry rotation time (s)	0.25
Pitch	1.4
Tube current	CareDose4D
Contrast material administration	50 mL contrast agent (Iomeron 400; Bracco) plus 50 mL NaCl at a rate of 3.5 mL/s (first phase: 80% contrast agent and 20% NaCl followed by saline flush)
Bolus tracking	Left ventricle
Threshold (HU)	100
Fixed trigger delay	
Inspiration (s)	10
Expiration (s)	300
Section thickness (mm)	1.0
Section interval (mm)	0.7
Convolution kernel at inspiration and expiration	
QR40	VNC and PBV
Br40	Monoenergetic
Br60	Lung

Photon-counting CT protocol used for simultaneous assessment of lung ventilation and perfusion. The protocol included both inspiratory and expiratory phases, along with intravenous contrast administration. Only ventilation parameters were evaluated in this study

### Image processing

**Fig. 1:**
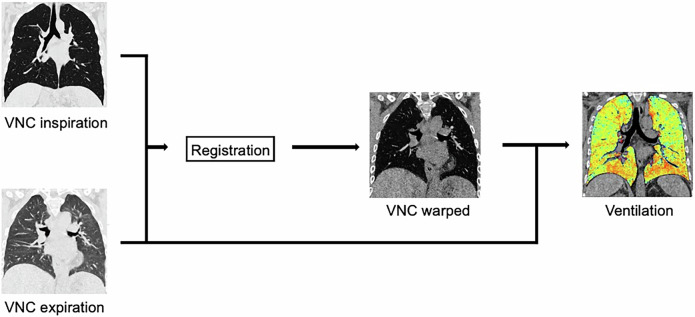
Image processing pipeline for CT-derived ventilation analysis. Inspiratory and expiratory scans were aligned using nonlinear image registration, and ventilation maps were generated by quantifying voxel-wise air volume changes based on attenuation differences between respiratory phases

CT-derived ventilation was calculated based on apparent volume changes in voxels between paired inspiratory and expiratory CT scans (Fig. 1). The following image processing steps were performed for this analysis:

1. **Lung segmentation**: Automatic lobar segmentation of inspiratory and expiratory virtual non-contrast images was performed using TotalSegmentator v2.7 [11] with standard settings and v2.5.0 weights. No retraining or fine-tuning was conducted.
2. **Image registration**: Inspiratory datasets were nonlinearly registered to expiratory datasets using open-source software (ANTs, release 2.1; Penn Image Computing and Science Laboratory) [12]. A three-stage registration framework was implemented, consisting of sequential rigid, affine, and nonlinear transformations to ensure progressively refined spatial alignment. Registration was performed in a multi-resolution (coarse-to-fine) scheme to improve robustness and convergence stability. For the linear stages (rigid and affine), mutual information (MI) was employed. The diffeomorphic nonlinear registration stage utilized the cross-correlation (CC) metric. To restrict the optimization to anatomically relevant regions, lung segmentation masks were incorporated throughout the registration process. Initialization was performed using a masked center-of-mass alignment, ensuring that the initial transformation was driven by lung parenchyma rather than surrounding background structures.
3. **Ventilation mapping**: Following registration, voxel-wise attenuation changes between phases were used as a surrogate for ventilation. Ventilation was expressed as the relative air volume change normalized to the inspiratory dataset:
  $${Ventilation}= -1000 * ({{{{\rm{HU}}}}}_{\exp }-{{{{\rm{HU}}}}}_{{{{\rm{insp}}}}})/ \\ ({{{{\rm{HU}}}}}_{{{{\rm{insp}}}}} * (1000+{{{{\rm{HU}}}}}_{\exp }))$$
  where *HU*_INSP_ and *HU*_EXP_ denote voxel attenuation at inspiration and expiration, respectively. Ventilation values were reported as percentages, ranging from 0% (no volume change) to 100% (complete collapse during expiration). Quantification was performed for the whole lung and individual lobes. The calculation was adapted from Simon et al [13], with a modification: in contrast to the original method, the inspiratory dataset served as the reference image instead of the expiratory dataset.
  Using the inspiratory dataset as the reference was intended to align the CT-derived ventilation metric with PFT parameters. In PFT, indices such as TLC, VC, and RV/TLC are defined relative to maximal inspiration (TLC). By normalizing voxel-wise air volume change to the inspiratory dataset, ventilation is expressed as the fraction of inspiratory lung volume exhaled, analogous to how VC or 1 − RV/TLC quantify air expelled from TLC.
4. **Regional analysis**: Each lung was divided into three equal coronal volumes (ventral, middle, dorsal) to assess gravitational gradients.

### Statistical analysis

Sample size estimation was based on expected TLC variability (16.2–17.1%) from published reference data [14]. Using a conservative 95% confidence interval and 5% tolerance error, the required sample size was 124 (62 per sex) [15]. Interim analyses indicated smaller-than-expected variability, and recruitment was concluded after 91 analyzable cases.

Data analysis was performed using JMPPro17 software version 18.0.2 (SAS Institute) and MATLAB R2024a (MathWorks). Continuous variables are presented as mean ± standard deviation, with 5th and 95th percentiles calculated to describe data distribution.

Normality was assessed with the Shapiro–Wilk test. Bland–Altman analysis quantified agreement between CT and PFT metrics, and Pearson’s correlation coefficients evaluated linear associations. Group differences by age, sex, height, regional distribution, and gravitational orientation were analyzed using paired *t*-tests and one-way ANOVA with Tukey correction (α = 0.05). Primary outcomes included lung volume, attenuation, mass, and ventilation, assessed for the whole lung and predefined subregions (lobes, gravity-dependent compartments, and attenuation-based subgroups). Lung parenchyma was categorized into

- Normal attenuation area (−950 Hounsfield units (HU) to −600 HU)
- High attenuation area (−599 HU to −250 HU)
- Very high attenuation area (> −250 HU)

A two-sided *p* < 0.05 was considered statistically significant.

## Results

### Participant characteristics

**Fig. 2:**
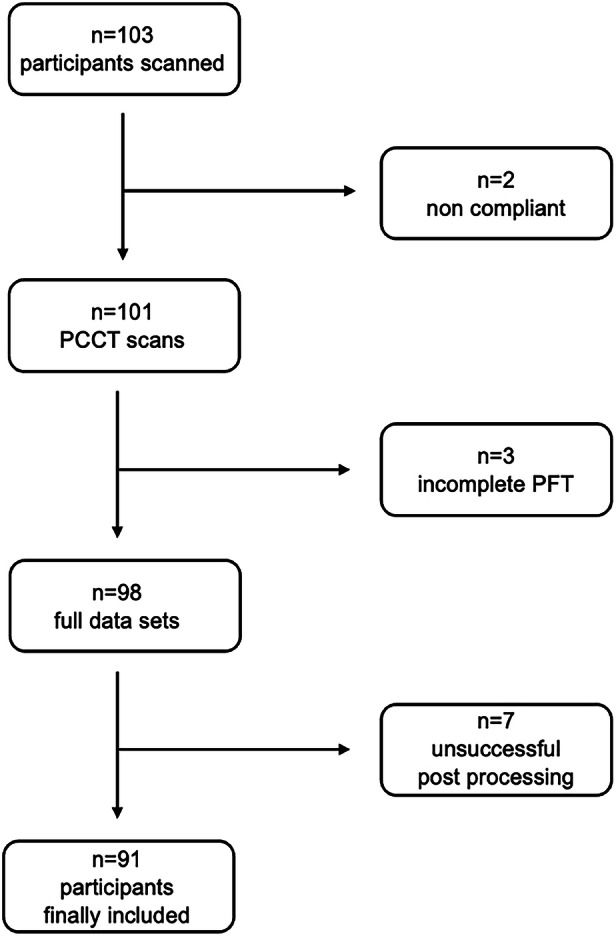
Flowchart illustrating the participant inclusion process for the study

The final study cohort included 91 participants (49 men (53.8%), 42 women (46.2%); mean age, 53 ± 12 years). Demographic characteristics are summarized in Table 2. Twelve individuals were excluded based on predefined criteria (Fig. 2).

**Table 2:** Demographic characteristics

	Male	Female	Total
*n*	49	42	91
Age (years)	53 ± 11.7	53 ± 11.5	53 ± 11.5
Height (cm)	180 ± 6.6	169 ± 6.3	175 ± 8.5
BMI (kg/m²)	27 ± 3.4	25 ± 3.7	26 ± 3.7

Demographic characteristics of the 91 participants included in the final analysis Data are expressed as mean ± standard deviation

### CT-derived ventilation reference values

Mean whole-lung ventilation was 59.5% ± 8.5% (5th–95th percentile, 42.1–72.5%).

### Global analysis

**Fig. 3:**
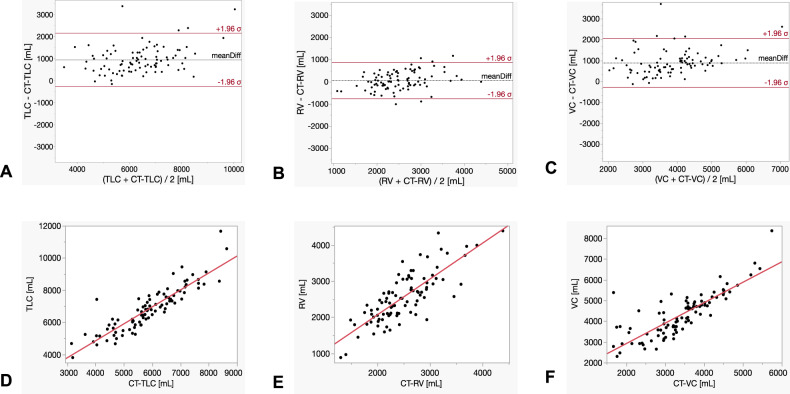
Bland–Altman analysis comparing PFT-derived and CT-derived lung volumes. Differences are defined as PFT − CT and plotted against the mean of both methods. The black dashed line represents the mean bias, and the red solid lines represent the 95% limits of agreement. **A** Total lung capacity: positive bias indicating CT underestimation. **B** Residual volume: minimal bias with good agreement. **C** Vital capacity: positive bias indicating CT underestimation. To estimate PFT values from CT-derived metrics, the following regression equations were obtained: **D** TLC = 1.0545 × CT-TLC + 625 mL (*r* = 0.89), **E** RV = 0.9898 × CT-RV + 89 mL (*r* = 0.80), and (**F**) VC = 0.9865 × CT-VC + 942 mL (*r* = 0.82)

CT- and PFT-derived lung volumes correlated strongly: CT-TLC (*r* = 0.89), CT-RV (*r* = 0.80), CT-VC (*r* = 0.82). CT systematically underestimated TLC and VC (*p* < 0.001) but not RV (*p* = 0.08) as can be seen on the Bland–Altman analyses (Fig. 3A–C) and with proportional differences increasing with lung size, total lung capacity, inspiratory volume, residual volume and expiratory volume (Table 3, Fig. 3D–F; Supplementary Table S1).

**Table 3:** Summary of pulmonary function test results and CT-derived lung metrics

Parameter	Mean ± SD	5th percentile	95th percentile
Total lung capacity (PFT) (mL)	6852 ± 1370	4737	9070
Total lung capacity (CT) (mL)	5906 ± 1162	3986	7863
Residual volume (PFT) (mL)	2555 ± 693	1568	3912
Residual volume (CT) (mL)	2492 ± 560	1599	3620
Vital capacity (PFT) (mL)	4310 ± 1053	2732	6183
Vital capacity (CT) (mL)	3414 ± 880	1810	5000
RV/TLC (PFT)	0.37 ± 0.08	0.25	0.50
RV/TLC (CT)	0.43 ± 0.07	0.32	0.59
Ventilation (%)	59.5 ± 8.5	42.1	72.5

Values are reported as mean ± standard deviation (SD) and the corresponding 5th and 95th percentiles. Parameters include residual volume (RV), total lung capacity (TLC), vital capacity (VC), and RV/TLC ratio from both pulmonary function tests (PFT) and CT, as well as relative ventilation. CT yielded systematically lower TLC and VC values and slightly lower RV compared with PFT, primarily reflecting the effects of supine positioning during CT versus upright measurements during PFT

Comparable correlation coefficients were obtained for same-day and delayed PFT measurements (mean delay, 44 days).

Both PFT- and CT-derived absolute lung volumes (residual volume (PFT: *p* = 0.001; CT: *p* < 0.001), total lung capacity (PFT and CT: *p* < 0.001), vital capacity (PFT and CT: *p* < 0.001)) were significantly lower in women than in men and positively correlated with height (for all values *r* > 0.38). Inspiratory volumes were not associated with age, whereas residual volume increased significantly with age based on PFT data (*p* = 0.003). The residual volume to total lung capacity (RV/TLC) ratio also increased with age (*p* < 0.002), independent of gender and height (Supplementary Tables S2–S4).

**Fig. 4:**
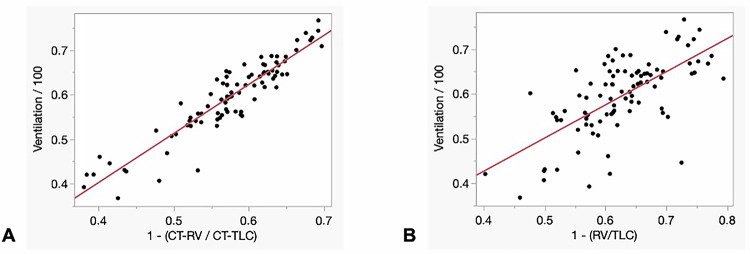
Correlation between CT-derived ventilation and relative vital capacity. **A** CT-based segmented lung volume ratio (1 − CT-RV/CT-TLC) demonstrated a strong correlation (*r* = 0.93). **B** PFT-derived 1 − RV/TLC showed a moderate correlation (*r* = 0.66)

CT-derived ventilation correlated moderately with relative vital capacity (1 − RV/TLC) in PFT (*r* = 0.66), whereas the correlation with the CT-based segmented lung volume ratio (1 − CT-RV/CT-TLC) was very strong (*r* = 0.93) (Fig. 4).

**Fig. 5:**
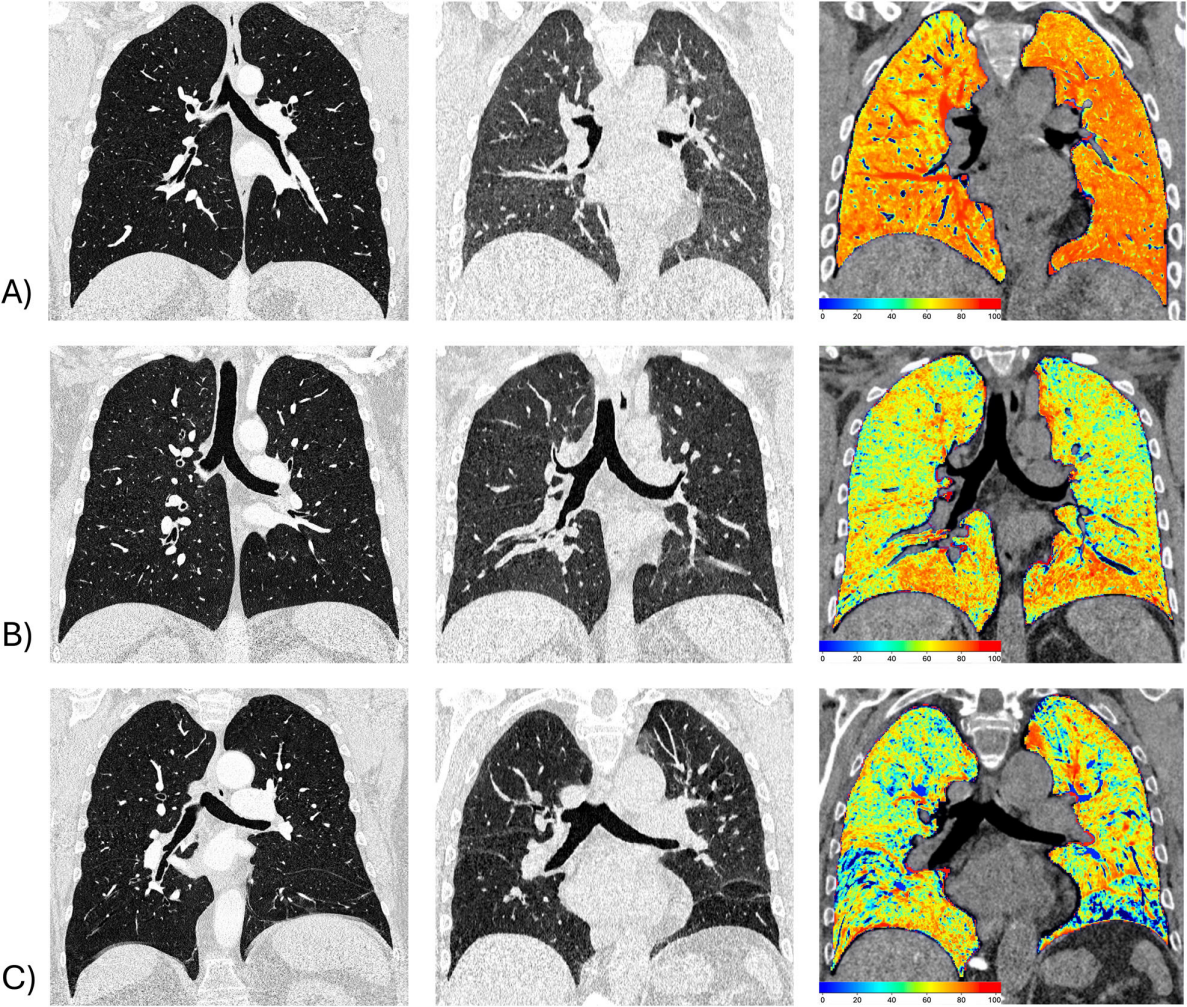
Coronal images obtained at inspiration (left), expiration (middle), and CT-derived ventilation maps (right) from three participants of different ages, demonstrating decreasing ventilation and increasing heterogeneity (increasing SD) with increasing age. **A** A 33-year-old participant demonstrating high and homogeneous ventilation of 76.6% ± 26.4%. **B** A 45-year-old participant showing moderate ventilation of 51.9% ± 23.2%. **C** A 76-year-old participant exhibiting reduced and heterogeneous ventilation of 40.7% ± 38.7%

**Fig. 6:**
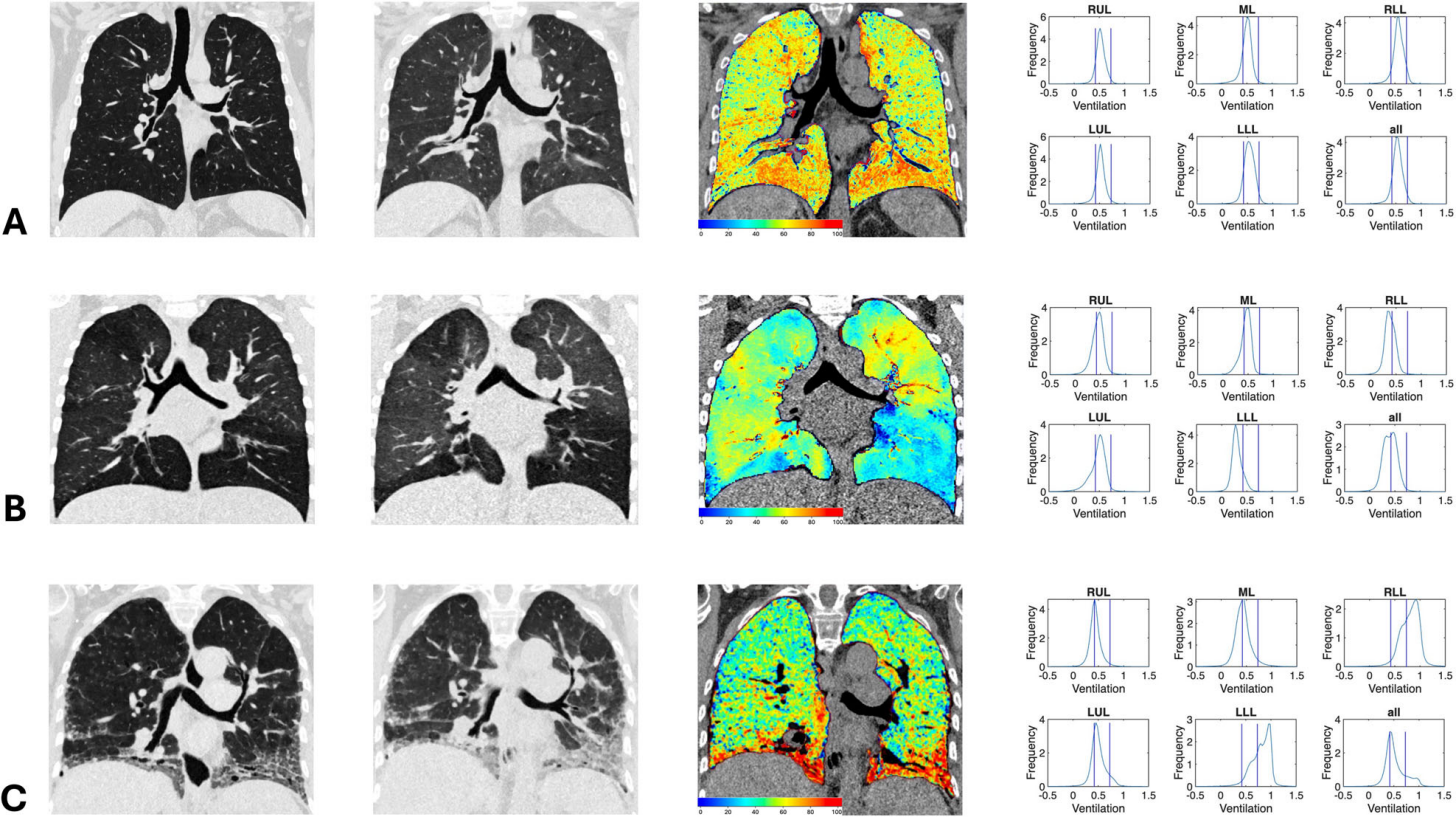
Coronal inspiratory (left) and expiratory (middle) CT images with corresponding CT-derived ventilation maps (right) and lobar ventilation histograms. Vertical lines represent the 5th and 95th percentiles. **A** Healthy subject from the study cohort with ventilation within the reference range. **B** Bronchiectasis with mucus plugging and extensive air trapping in the lower lobes (illustrative, not from study cohort). **C** Pulmonary fibrosis with a UIP pattern in silicosis, showing subpleural reticulation and alveolar collapse with pathologically increased lower-lobe ventilation (illustrative, not from study cohort)

Opposed to lung volumes, no significant sex differences were observed in ventilation. Ventilation metrics showed only a weak correlation with height (*r* = 0.13) and declined with age (< 50 vs ≥ 60 years, *p* < 0.001; 50–59 vs ≥ 60 years, *p* = 0.004). Representative ventilation maps of healthy subjects are presented in Fig. 5. Illustrative pathological examples from independent clinical cases not enrolled in the study and not included in the analysis are presented in Fig. 6.

### Regional ventilation distribution

Ventilation exhibited pronounced regional heterogeneity across lobes, tissue density classes, and gravitational orientations.

### Lobar distribution

Lower lobes demonstrated significantly higher ventilation than upper lobes, with marked differences between the right upper lobe and both lower lobes (*p* < 0.001 vs right lower lobe; *p* = 0.003 vs left lower lobe; Table 4). Consistently, mean expiratory attenuation was higher in the lower than in the upper lobes (*p* < 0.001; Supplementary Table S5). The right middle lobe showed significantly lower ventilation than all other lobes (*p* < 0.001).

**Table 4:** Respiratory parameters across different lung lobes

	RUL	ML	RLL	LUL	LLL
Expirational lung volume (mL)	407 ± 192	173 ± 84	582 ± 161	568 ± 152	525 ± 129
Inspirational lung volume (mL)	1109 ± 227	472 ± 121	1581 ± 344	1331 ± 253	1412 ± 352
Expiration (vol)/inspiration (vol)	0.37 ± 0.16	0.37 ± 0.16	0.37 ± 0.08	0.43 ± 0.08	0.38 ± 0.08
Inspiration (vol) − expiration (vol) (mL)	703 ± 236	299 ± 116	999 ± 268	764 ± 189	887 ± 287
Inspirational lung volume distribution (%)	18.8 ± 1.7	8.0 ± 1.3	26.7 ± 2.0	22.7 ± 2.1	23.8 ± 2.4
Ventilation (%)	57.3 ± 8.8	49.9 ± 10.2	63.0 ± 9.0	58.8 ± 8.5	62.4 ± 9.7
Ventilation distribution (%)	18.2 ± 2.5	6.7 ± 1.4	28.3 ± 2.5	22.5 ± 2.5	25.0 ± 3.3

The table illustrates key respiratory trends. The consistency in lung volume ratios suggested uniform respiratory dynamics, while the lower lobes demonstrated higher ventilation values. Results for lobar mass and mean attenuation can be found in Supplementary Table S6

*RUL* right upper lobe, *ML* right middle lobe, *RLL* right lower lobe, *LUL* left upper lobe, *LLL* left lower lobe

In terms of relative contributions to total ventilation, the right and left lower lobes accounted for 28.3% and 25.0% of total ventilation, respectively, followed by the left upper lobe (22.5%) and right upper lobe (18.2%). The right middle lobe contributed only 6.7%. Corresponding percentile ranges (5th–95th) are summarized in Supplementary Table S5.

### Tissue density

Ventilation strongly depended on parenchymal density. Normal attenuation areas exhibited the highest and most homogeneous ventilation (63.1% ± 8.9%). Regions with increased attenuation showed markedly reduced ventilation (–0.5% ± 17.6%), while very high attenuation areas demonstrated further impairment (–4.9% ± 3.9%), including negative values indicative of paradoxical or non-functional airflow (Supplementary Table S6).

### Gravity dependence

Ventilation followed a gravity-dependent pattern, with significantly higher values in dorsal (60.5% ± 10.1%) compared with ventral lung regions (48.5% ± 11.3%; *p* < 0.001) (Supplementary Table S7, Supplementary Fig. S1).

## Discussion

This study provides initial reference data on regional pulmonary ventilation in healthy individuals, addressing the broader lack of standardized CT-based benchmarks needed to identify disease-related deviations in functional lung imaging.

CT-based quantification of lung volumes is well established and shows good correlation with plethysmographic measurements. However, most available CT reference equations focus on static global volumes rather than regional ventilation metrics. For example, Bakker et al [16]. provide reference formulas for chest CT-derived lobar volumes in a lung-healthy population, underscoring the feasibility of CT-based normative modeling but highlighting that these data relate primarily to volumetry rather than functional ventilation indices.

Registration-based CT ventilation approaches—including density change, specific volume change, and Jacobian-based metrics—have demonstrated correlation with spirometric and plethysmographic parameters, particularly in chronic obstructive pulmonary disease populations, and can reliably reflect functional impairment in diseased lungs [17]. Several studies confirm reproducibility and related metrics in healthy volunteers [18, 19], yet percentile-based normal ranges are not reported. Similarly, deformation-based analyses [20] confirmed agreement between Jacobian-derived and segmentation-based global volume changes, with abnormality typically defined using percentile thresholds (e.g., lowest 20–25%) instead of formal reference intervals. Most available work focuses on methodological validation, reproducibility, or disease discrimination rather than establishing population-based standards for regional ventilation.

For interpreting regional ventilation abnormalities across a range of pulmonary diseases—including obstructive, restrictive, vascular, and post-transplant conditions [10, 21]—normative values are essential and may support more individualized disease assessment and treatment monitoring. CT-based methods offer the unique capability to regionally relate functional impairment directly to structural abnormalities, enhancing the understanding of structure–function relationships. Although the study was performed on a photon-counting CT system, the underlying methodology is not detector-specific and is applicable across modern CT platforms. Establishing normative values and relating these measurements to established physiologic standards, such as pulmonary function tests (PFTs), remain critical step toward broader clinical implementation [22].

This work provides an initial analysis of regional ventilation values derived from a standardized CT-based process and demonstrates moderate to strong correlations with PFT parameters. CT-based estimates of total lung capacity (TLC) and vital capacity (VC) were systematically lower than PFT-derived values, whereas residual volume (RV) was comparable. These offsets likely reflect differences in acquisition conditions—supine positioning during CT versus upright during PFT—which influence thoracic mechanics and reduce inspiratory capacity, a well-documented effect in comparative pulmonary function studies [15].

Despite these systematic differences, the strong correlations and relatively narrow 95% limits of agreement indicate that the relationship between CT-derived and PFT-derived measurements is predictable, enabling intermodality comparison and supporting the use of CT-derived ventilation metrics as quantitative imaging biomarkers.

However, CT- and PFT-derived values should not be considered interchangeable in absolute terms, as they reflect related but not identical physiological constructs—regional imaging-based lung volumes acquired under static breath-hold conditions versus global plethysmographic measurements under standardized upright testing.

For this reason, CT-derived parameters should be interpreted within CT-specific normative reference ranges rather than using PFT reference equations. The present study provides such modality-specific benchmarks, enabling standardized interpretation of ventilation and lung volume metrics within the imaging domain.

The principal advantage of CT-based ventilation assessment is its capacity to provide spatially resolved quantification of regional lung function and to directly link functional impairment to underlying structural abnormalities. Moreover, it enables identification of regions at risk for future structural remodeling, as functional alterations frequently precede morphologic changes—capabilities that have no direct counterpart in conventional pulmonary function testing.

Although the variations in segmented lung volumes during inhalation and exhalation closely corresponded with lobar-level ventilation measurements (Table 4), a central challenge in CT-based functional imaging remains the absence of a true reference standard for regional ventilation. Because ventilation cannot be measured directly with CT, various surrogate metrics—such as voxel-wise attenuation changes or deformation-based volume differences—have been proposed [10, 23–26].

We therefore compared three methodological approaches: (1) attenuation-based calculations, (2) deformation-based analysis using the inverse Jacobian determinant, and (3) a combined method incorporating both. Each approach has conceptual advantages and limitations, but all yielded similar mean ventilation values and variance [27].

A detailed methodological comparison was beyond the scope of this work. For the present analysis, we selected an attenuation-based method due to its conceptual alignment with established CT biomarkers such as parametric response mapping (PRM) in emphysema and small-airway disease quantification [28].

Based on our normative distribution, expiratory attenuation thresholds associated with air trapping (–856 HU) [29] and alveolar collapse (–250 HU) [30] fall below the 1st percentile and above the 99th percentile, respectively, underscoring the utility of these values for contextualizing disease-related abnormalities.

Demographic analyses revealed minimal effects of sex and height on ventilation metrics, while age showed a mild positive association with RV, likely reflecting age-related reductions in elastic recoil. However, the current cohort was not large enough to derive robust age- or sex-specific reference equations, and the potential influence of ethnicity requires validation in broader, multicenter populations. Lobar and gravitational ventilation gradients followed established physiological patterns, with higher ventilation in dependent regions and consistently lower values in the right middle lobe [31, 32]. These findings support the sensitivity of the CT-based process to physiological ventilation heterogeneity and its potential to detect subtle regional abnormalities. Understanding physiological regional ventilation patterns is essential for interpreting disease-related abnormalities. The identified lobar and gravity-dependent gradients provide a normative reference to distinguish normal heterogeneity from pathology. In chronic obstructive pulmonary disease, ventilation deficits exceeding expected regional differences may indicate focal obstruction, whereas in interstitial lung disease, altered regional ventilation can reflect compensatory redistribution or fibrosis-related impairment. Defining normal variability therefore improves diagnostic accuracy and sensitivity for subtle disease-specific changes.

Accordingly, standardized CT-derived ventilation mapping has potential clinical relevance across multiple disease domains, including chronic obstructive pulmonary disease, fibrotic interstitial lung disease, pulmonary hypertension, and post-transplant evaluation. Quantification of regional ventilation may enhance disease characterization, refine phenotyping, and support treatment monitoring [30].

To illustrate clinical applicability, we included examples from two patients outside the study cohort—one with predominantly obstructive and one with restrictive ventilatory impairment (Fig. 6).

This study has several limitations. Although the cohort was sufficiently large to establish initial reference intervals, larger and more diverse populations are needed to develop demographic-specific reference equations and validate generalizability. Whilst the mean BMI of 26 in our cohort is slightly above the conventional “normal” range (18.5–24.9 kg/m²), it reflects the BMI distribution commonly observed in contemporary adult populations. The cohort also exhibits expected demographic patterns, including higher BMI values in men than in women. Regarding functional impact, increased BMI may influence ventilation metrics through reduced chest wall compliance, altered diaphragmatic excursion, and mild reductions in expiratory reserve volume. However, in our analysis, ventilation showed only weak associations with anthropometric parameters, suggesting that within the studied BMI range, body composition did not exert a dominant effect on CT-derived ventilation.

Precise voxel-wise alignment of inspiratory and expiratory scans remains technically challenging, particularly in expiration, and may influence local ventilation estimates. We did not assess repeatability because this would require duplicate CT examinations in healthy volunteers. However, prior work in a porcine model using similar processing showed high scan–rescan agreement [33]. Furthermore, Shin et al [34] demonstrated high intraclass correlation coefficients and within-subject variability typically below 10% for consecutive inspiratory scans, even without spirometric gating. Large-cohort analyses and reviews [35] further identified inspiratory level as one of the dominant contributors to variance in density-based QCT measures and emphasized that variability can be substantially reduced through lung volume control (breath coaching, spirometric gating, or mathematical adjustment).

In our study, spirometry-guided breath-hold acquisition and structured breathing training were implemented to minimize variability in inspiratory and expiratory lung volumes. The strong correlations between CT-derived and PFT-derived volumes additionally argue against excessive random variability in our dataset.

Attenuation values were derived from virtual non-contrast images because contrast material was administered for parallel perfusion measurements. Prior studies and our own validation indicate that virtual non-contrast-based attenuation is reliable for functional mapping [36, 37]. The small systematic difference between virtual non-contrast and true non-contrast images (mean bias, 1.1 ± 5.9 HU) was negligible compared with the variability introduced by differences in inspiratory depth (mean, 2.7 ± 18.1 HU), highlighting the robustness of virtual non-contrast-based ventilation estimation (unpublished data).

In summary, this standardized CT-based workflow enables robust, spatially resolved quantification of regional pulmonary ventilation in healthy adults and shows strong correspondence with global PFT measures. The resulting reference values—though derived from a single-center cohort scanned on a photon-counting CT system—are intended to be applicable across CT platforms. These data provide a foundational step toward evaluating CT-derived ventilation as a functional imaging biomarker in obstructive and restrictive lung disease and support the development of automated analysis pipelines and longitudinal prognostic studies.

## Supplementary information

ELECTRONIC SUPPLEMENTARY MATERIAL

## Abbreviations

HU: Hounsfield units
PFT: Pulmonary function test
RV: Residual volume
TLC: Total lung capacity
VC: Vital capacity
