# Supplementary material for: CT-based lung ventilation metrics: reference ranges and pulmonary function test correlations in healthy individuals
Source: Eur Radiol. 2026 Apr 28;36(9):7152–63. doi: 10.1007/s00330-026-12559-8 (PMC13451287; doi:10.1007/s00330-026-12559-8)
Supplement: Supplementary file 1 [file 330_2026_12559_MOESM1_ESM.pdf]

CT-Based Lung Ventilation Metrics: Reference Ranges and Pulmonary Function Test Correlations in Healthy Individuals

ELECTRONIC SUPPLEMENTARY MATERIAL

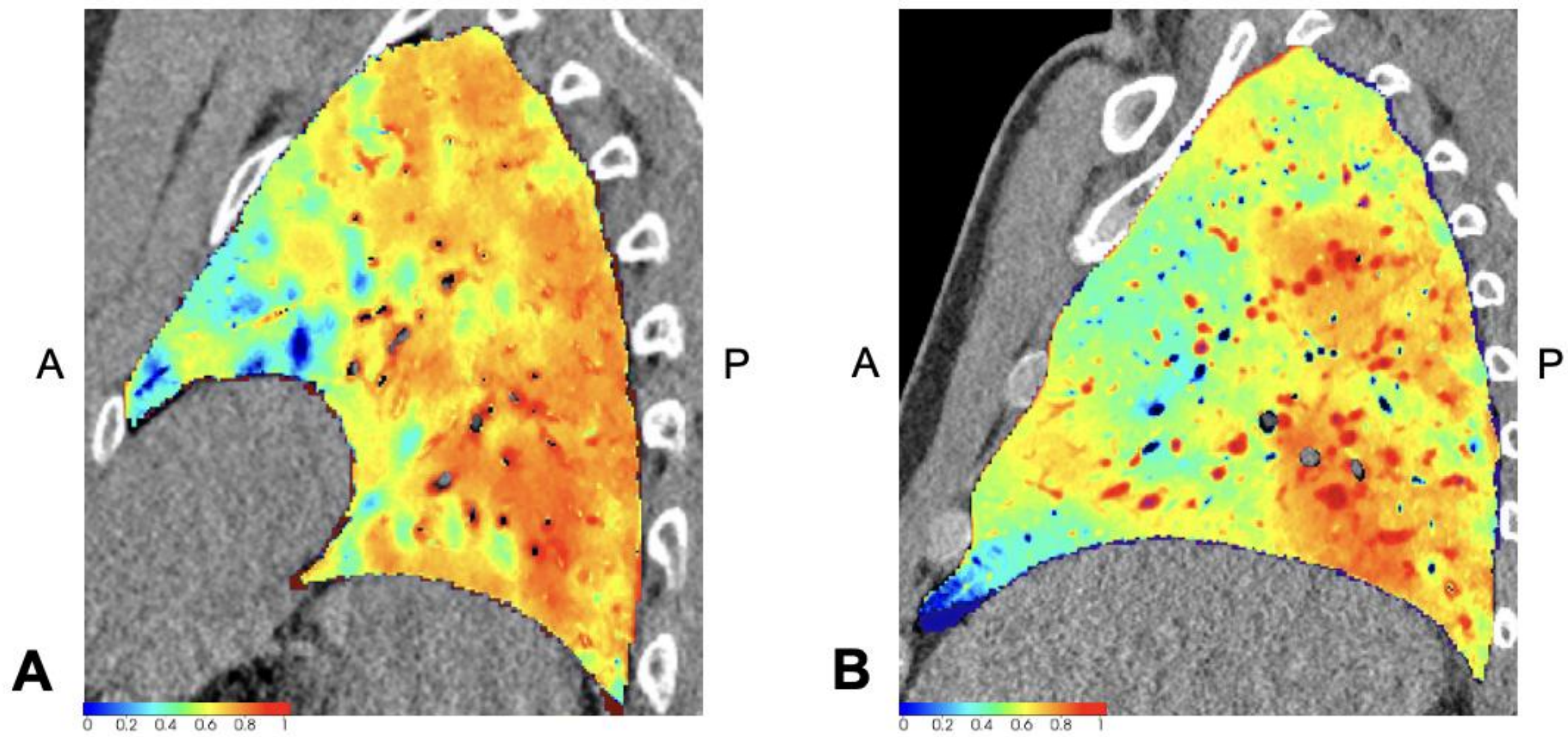

Figure S1. Spatial ventilation gradients across the left (A) and right (B) lungs. Ventilation increased from anterior to posterior regions, demonstrating gravity-dependent airflow distribution in the supine position.

	CT	PFT	difference	r	P
Total Lung Capacity (TLC) [mL]	5906 +-1162 [3986; 7863]	6852 +-1370 [4737; 9070]	947 +-616 [190; 2075]	0.89	**< .001**
Residual Volume (RV) [mL]	2492 +-560 [1599; 3620]	2555 +-693 [1568; 3912]	63 +-416 [-636; 744]	0.80	.075
Vital Capacity (VC) [mL]	3414 +-880 [1810; 5000]	4310 +-1053 [2732; 6183]	896 +-597 [133; 2091]	0.82	**< .001**
RV / TLC	0.43 +-0.07 [0.32; 0.59]	0.37 +-0.08 [0.25; 0.50]	0.05 +-0.07 [-0.04; 0.19]	0.61	**< .001**

Table S1. Comparison of lung volume measurements obtained using CT and conventional pulmonary function testing (PFT).

Values are reported as mean ± standard deviation (SD), with the 5th and 95th percentiles indicated in brackets. The "difference" column denotes the mean difference between CT- and PFT-derived measurements. Agreement between the two modalities is quantified using Pearson’s correlation coefficient (r). P-values were obtained from two-sided paired t-tests.

	w (n=42)	m (n=49)	P-value
Residual Volume (PFT) [mL]	2304 +-598	2770 +-702	**.001**
Residual Volume (CT) [mL]	2200 +-424	2742 +-543	**< .001**
Total Lung Capacity (PFT) [mL]	5891 +-850	7676 +-1184	**< .001**
Total Lung Capacity (CT) [mL]	5116 +-887	6582 +-921	**< .001**
Vital Capacity (PFT) [mL]	3589 +-699	4927 +-906	**< .001**
Vital Capacity (CT) [mL]	2916 -+-702	3840 +-792	**< .001**
RV / TLC (PFT)	0.39 +-0.08	0.36 +-0.07	.072
CT-RV / CT-TLC (CT)	0.43 +-0.07	0.42 +-0.07	.323
Expirational lung mass [g]	696 +-95	863 +-123	**< .001**
Inspirational lung mass [g]	735 +-102	917 +-127	**< .001**
Expiration(mass) / Inspiration(mass)	0.95 +-0.04	0.94 +-0.05	.482
Expirational mean attenuation [HU]	-676 +-54	-678 +-54	.873
Inspirational mean attenuation [HU]	-854 +-22	-859 +-19	.205
Expiration(HU) / Inspiration(HU)	0.79 +-0.05	0.79 +-0.06	.836
Ventilation [%]	59.7 +-9.0	59.1 +-8.0	.746

Table S2. Subgroup analysis based on gender. Generally, males have higher residual volumes, total lung capacity, and vital capacity than females. Inspiratory and expiratory volumes, as well as lung mass, are also greater in males. Ratios of expiration to inspiration and mean attenuation values are similar across genders, and ventilation percentages show only minimal variation. P-values were obtained from two-sided t-tests (α = 0.05).

	< 50 years (n=30)	50-59 years (n=36)	> 59 years (n=25)
Residual Volume (PFT) [mL]	2208 +-730	2668 +-537	2810 +-705
Residual Volume (CT) [mL]	2314 +-659	2518 +-451	2667 +-530
Total Lung Capacity (PFT) [mL]	6890 +-1692	7012 +-1076	6577 +-1332
Total Lung Capacity (CT) [mL]	5850 +-1527	6048 +-877	5766 +-1035
Vital Capacity (PFT) [mL]	4683 +-1212	4374 +-791	3769 +-995
Vital Capacity (CT) [mL]	3536 +-1132	3531 +-657	3099 +-763
RV / TLC (PFT)	0.32 +-0.07	0.38 +-0.05	0.43 +-0.08
CT-RV / CT-TLC (CT)	0.40 +-0.08	0.42 +-0.05	0.47 +-0.07
Expirational lung mass [g]	812 +-160	787 +-125	752 +-127
Inspirational lung mass [g]	850 +-161	835 +-138	809 +-147
Expiration(mass) / Inspiration(mass)	0.95 +-0.04	0.94 +-0.05	0.93 +-0.04
Expirational mean attenuation [HU]	-640 +-49	-684 +-39	-713 +-50
Inspirational mean attenuation [HU]	-850 +-26	-861 +-16	-859 +-17
Expiration(HU) / Inspiration(HU)	0.75 +-0.06	0.79 +-0.04	0.83 +-0.05
Ventilation [%]	63.8 +-8.4	60.0 +-6.5	53.5 +-8.1

Table S3. Subgroup analysis based on age. With age, there is an increase in residual volume and the RV/TLC ratio. Total lung capacity and vital capacity peak during middle age (ages 50–59) and then decline. Inspiratory and expiratory volumes, as well as lung mass, exhibit similar trends. Older participants (>59 years) show lower values than younger groups. Ventilation efficiency decreases in the oldest group. With increasing age, mean lung attenuation decreases ($P < .05$). Group differences by age were evaluated using one-way ANOVA with Tukey post hoc correction ($\alpha = 0.05$).

	< 170 cm (n=27)	170-180 cm (n=37)	181-200 cm (n=27)
Residual Volume (PFT) [mL]	2158 +-565	2699 +-721	2755 +-622
Residual Volume (CT) [mL]	2172 +-496	2555 +-561	2725 +-483
Total Lung Capacity (PFT) [mL]	5551 +-818	7039 +-1012	7897 +-1214
Total Lung Capacity (CT) [mL]	4884 +-922	6089 +-775	6675 +-1117
Vital Capacity (PFT) [mL]	3396 +-690	4343 +-706	5177 +-1012
Vital Capacity (CT) [mL]	2712 +-662	3535 +-554	3950 +-992
RV / TLC *(PFT)*	0.39 +-0.09	0.38 +-0.07	0.35 +-0.07
CT-RV / CT-TLC (CT)	0.45 +-0.07	0.42 +-0.06	0.42 +-0.08
Expirational lung mass [g]	677 +-106	777 +-109	907 +-107
Inspirational lung mass [g]	715 +-111	829 +-116	957 +-118
Expiration(mass) / Inspiration(mass)	0.95 +-0.04	0.94 +-0.05	0.95 +-0.04
Expirational mean attenuation [HU]	-678 +-62	-688 +-50	-661 +-47
Inspirational mean attenuation [HU]	-851 +-24	-863 +-13	-854 +-23
Expiration(HU) / Inspiration(HU)	0.80 +-0.06	0.80 +-0.05	0.77 +-0.06
Ventilation [%]	58.1 +-8.2	59.2 +-8.4	61.1 +-9.2

Table S4. Subgroup analysis based on height.

Taller statures are associated with increased residual volume, total lung capacity, and vital capacity. Participants over 180 cm have higher inspiratory and expiratory volumes and lung mass. The RV/TLC ratio and attenuation values show slight variations across heights, indicating consistent ventilation efficiency and highlighting the physical differences in lung capacity associated with height. A positive correlation was observed between height and lung mass, while the expiratory-to-inspiratory lung mass ratio remained stable. Group differences by height were evaluated using one-way ANOVA with Tukey post hoc correction ($\alpha = 0.05$).

	RUL	ML	RLL	LUL	LLL
Expirational lung volume [mL]	407 +-192 [87; 688]	173 +-84 [43; 320]	582 +-161 [314; 872]	568 +-152 [331; 863]	525 +-129 [335; 775]
Inspirational lung volume [mL]	1109 +-227 [722; 1553]	472 +-121 [273; 652]	1581 +-344 [975; 2098]	1331 +-253 [926; 1761]	1412 +-352 [798; 2049]
Expiration(vol) / Inspiration(vol)	0.37 +-0.16 [0.08; 0.59]	0.37 +-0.16 [0.10; 0.63]	0.37 +-0.08 [0.23; 0.55]	0.43 +-0.08 [0.31; 0.60]	0.38 +-0.08 [0.27; 0.54]
Inspirational lung volume distribution [%]	18.8 +-1.7 [15.9; 22.0]	8.0 +-1.3 [5.7; 10.3]	26.7 +-2.0 [23.3; 29.9]	22.7 +-2.1 [19.5; 26.7]	23.8 +-2.4 [18.7; 26.6]
Expirational lung mass [g]	118 +-51 [23; 204]	44 +-20 [9; 77]	200 +-46 [120; 278]	169 +-34 [115; 229]	184 +-38 [122; 256]
Inspirational lung mass [g]	152 +-30 [110; 212]	63 +-16 [39; 90]	227 +-41 [159; 305]	183 +-35 [125; 240]	208 +-41 [139; 291]
Expiration(mass) / Inspiration(mass)	0.77 +-0.29 [0.15; 1.03]	0.70 +-0.26 [0.20; 0.96]	0.88 +-0.11 [0.58; 1.00]	0.92 +-0.08 [0.80; 1.01]	0.89 +-0.06 [0.79; 0.99]
Inspirational lung mass distribution [%]	18.3 +-1.5 [15.9; 20.9]	7.5 +-1.1 [5.5; 9.4]	27.2 +-1.7 [24.6; 29.9]	22.0 +-1.7 [18.8; 24.8]	24.9 +-2.0 [21.2; 27.8]
Expirational mean [HU]	-700 +-56 [-777; -584]	-737 +-56 [-810; -646]	-646 +-60 [-760; -551]	-693 +-56 [-775; -583]	-641 +-63 [-742; -531]
Inspirational mean [HU]	-861 +-19 [-884; -819]	-865 +-16 [-884; -829]	-854 +-24 [-879; -805]	-861 +-18 [-883; -816]	-849 +-27 [-877; -791]
Expiration(HU) / Inspiration(HU)	0.81 +-0.06 [0.71; 0.91]	0.85 +-0.06 [0.77; 0.93]	0.76 +-0.06 [0.66; 0.90]	0.80 +-0.06 [0.69; 0.91]	0.75 +-0.07 [0.64; 0.88]
Ventilation [%]	57.3 +-8.8 [40.1; 70.4]	49.9 +-10.2 [30.0; 65.7]	63.0 +-9.0 [42.9; 74.8]	58.8 +-8.5 [42.0; 73.0]	62.4 +-9.7 [41.9; 74.4]
Ventilation distribution [%]	18.2 +-2.5 [14.2; 22.9]	6.7 +-1.4 [4.3; 9.1]	28.3 +-2.5 [23.5; 32.7]	22.5 +-2.5 [18.5; 27.6]	25.0 +-3.3 [18.7; 29.7]

Table S5. Distribution of respiratory parameters across lung lobes, including 5th and 95th percentiles in brackets. RUL: right upper lobe; ML: right middle lobe; RLL: right lower lobe; LUL: left upper lobe; LLL: left lower lobe.

	NAA	HAA	VHAA
Expirational lung volume [mL]	2349 +-534 [1481; 3421]	102 +-27 [65; 154]	41 +-11 [26; 62]
Inspirational lung volume [mL]	5611 +-1126 [3715; 7486]	224 +-44 [158; 310]	71 +-13 [47; 96]
Expiration(vol)/(Inspiration(vol)	0.42 +-0.07 [0.31; 0.58]	0.45 +-0.08 [0.34; 0.62]	0.58 +-0.11 [0.41; 0.78]
Lung volume distribution [%]	94.9 +-0.9 [92.8; 95.9]	3.9 +-0.8 [3.0; 5.4]	1.2 +-0.2 [1.0; 1.7]
Expirational lung mass [g]	697 +-126 [499; 937]	58 +-17 [35; 93]	30 +-10 [17; 50]
Inspirational lung mass [g]	651 +-117 [460; 855]	121 +-24 [86; 167]	61 +-12 [41; 82]
Expiration(mass)/Inspiration(mass)	1.07 +-0.06 [0.98; 1.16]	0.48 +-0.10 [0.32; 0.67]	0.50 +-0.13 [0.30; 0.72]
Lung mass distribution [%]	78.1 +-1.5 [75.4; 80.0]	14.6 +-1.2 [13.1; 16.8]	7.3 +-0.7 [6.3; 8.5]
Ventilation [%]	63.1 +-8.9 [44.2; 76.5]	-0.5 +-17.6 [-29.8; 30.0]	-4.9 +-3.9 [-12.1; 1.1]
Ventilation distribution [%]	100.8 +-1.1 [98.5; 102.3]	0.0 +-1.2 [-1.8; 2.4]	-0.1 +-0.1 [-0.2; 0.0]

Table S6. Effects of tissue density on respiratory parameters.

Highly attenuated areas (HAA) and very highly attenuated areas (VHAA) comprised only a small fraction of the total lung volume and mass. However, these regions were associated with increased variability in the expiration-to-inspiration volume ratio, reflecting altered ventilation dynamics.

Parameter	lower	middle	upper
Expirational mean [HU]	-634 +-67 [-756; -523]	-681 +-54 [-772; -568]	-758 +-47 [-832; -670]
Inspirational mean [HU]	-838 +-30 [-878; -776]	-844 +-24 [-875; -790]	-868 +-19 [-896; -824]
Expiration(HU) / Inspiration(HU)	0.76 +-0.07 [0.65; 0.89]	0.81 +-0.06 [0.72; 0.90]	0.87 +-0.05 [0.80; 0.94]
Ventilation [%]	60.5 +-10.1 [42.2; 75.9]	56.1 +-9.5 [40.8; 70.6]	48.5 +-11.3 [29.4; 65.4]

Table S7. Effects of gravity on respiratory parameters. Dependent lung regions show higher ventilation and attenuation values compared to non-dependent regions.
